# Simulation Study of CO_2_-EOR in Tight Oil Reservoirs with Complex Fracture Geometries

**DOI:** 10.1038/srep33445

**Published:** 2016-09-15

**Authors:** Pavel Zuloaga-Molero, Wei Yu, Yifei Xu, Kamy Sepehrnoori, Baozhen Li

**Affiliations:** 1 Department of Petroleum and Geosystems Engineering, University of Texas at Austin, Austin, TX, 78712, USA; 2 Department of Petroleum Engineering, Texas A&M University, College Station, TX, 77843, USA; 3 CNOOC Research Institute, Beijing, 100027, China

## Abstract

The recent development of tight oil reservoirs has led to an increase in oil production in the past several years due to the progress in horizontal drilling and hydraulic fracturing. However, the expected oil recovery factor from these reservoirs is still very low. CO_2_-based enhanced oil recovery is a suitable solution to improve the recovery. One challenge of the estimation of the recovery is to properly model complex hydraulic fracture geometries which are often assumed to be planar due to the limitation of local grid refinement approach. More flexible methods like the use of unstructured grids can significantly increase the computational demand. In this study, we introduce an efficient methodology of the embedded discrete fracture model to explicitly model complex fracture geometries. We build a compositional reservoir model to investigate the effects of complex fracture geometries on performance of CO_2_ Huff-n-Puff and CO_2_ continuous injection. The results confirm that the appropriate modelling of the fracture geometry plays a critical role in the estimation of the incremental oil recovery. This study also provides new insights into the understanding of the impacts of CO_2_ molecular diffusion, reservoir permeability, and natural fractures on the performance of CO_2_-EOR processes in tight oil reservoirs.

Tight oil reservoirs are typically characterized by low porosity (<10%) and low permeability (<0.1 mD)^1^. The successful economic development of tight oil reservoirs in recent years hinges on two advanced technologies: horizontal drilling and multi-stage hydraulic fracturing. U.S. Energy Information Administration^2^ reported that tight oil production will increase from 33% of total lower 48 onshore oil production to 51% in 2040. However, the decline curves of primary production are steep due to low permeability.

The Bakken Field located in North Dakota, is one the most productive tight oil reservoirs in North America. The Bakken formation is composed of shale and dolomite layers. The Middle Bakken and Three Forks layers are the most important because of the reservoir quality and hydrocarbon saturation. The primary oil recovery factor in Bakken Formation is very low and typically less than 10%, resulting in tremendous oil resource remaining in place. Hawthorne *et al*.^3^ presented that a minor improvement in oil recovery factor such as 1% could yield several billion barrels of additional oil. Hence, it is important to investigate enhanced oil recovery (EOR) methods to increase oil recovery in tight oil reservoirs.

It is challenging to apply water flooding in tight oil reservoir due to low injectivity and poor sweep efficiency. Also, the oil-wet nature of some tight oil formations such as Bakken minimizes the effectiveness of water flooding. Both recent experimental and simulation studies have shown that carbon dioxide (CO_2_) injection could be a feasible EOR method to improve the oil recovery and carbon storage and sequestration in tight oil reservoirs^4^,^5^,^6^,^7^,^8^,^9^. CO_2_ has a considerably lower minimum miscibility pressure (MMP) than other gases such as N_2_ and CH_4_^10^,^11^. CO_2_-EOR has two common operation scenarios: continuous CO_2_ injection, which is referred to as CO_2_ flooding in this study, and CO_2_ Huff-n-Puff. Although CO_2_-EOR in conventional reservoir is well understood, it is relatively a new concept in tight oil reservoirs^1^. Hawthorne *et al*.^3^ proposed five conceptual steps for CO_2_-EOR process in tight oil formation: “(1) CO_2_ flows into and through the fractures, (2) unfractured rock matrix is exposed to CO_2_ at fracture surfaces, (3) CO_2_ permeates the rock driven by pressure, carrying some hydrocarbon inward; however, the oil is also swelling and extruding some oil out of the pores, (4) oil migrates to the bulk CO_2_ in the fractures via swelling and reduced viscosity, and (5) as the CO_2_ pressure gradient gets smaller, oil production is slowly driven by concentration gradient diffusion from pores into the bulk CO_2_ in the fractures”. The CO_2_ molecular diffusivity is a key physical mechanism for CO_2_-EOR process in tight oil reservoirs, which must be taken into account correctly when building a numerical compositional model^12^,^13^.

An accurate modeling CO_2_-EOR process in tight oil reservoirs is challenging. The actual hydraulic fracturing treatment often creates complex fracture networks by opening and interconnecting the pre-existing natural fractures. The fracture diagnostic technologies like microseismic^14^,^15^,^16^ and the recent developed fracture propagation models^17^,^18^,^19^ indicate that the fracture geometry is complex and non-planar. Although many efforts in the literature are dedicated to modeling CO_2_-EOR in tight oil reservoirs^20^,^21^,^22^,^23^, they made an assumption of simple bi-wing planar fracture geometry without considering the more-realistic complex fracture geometry. Even though the complex fracture networks provide the primary conduits for oil production, they might be detrimental to CO_2_ flooding effectiveness^24^. This is because of early CO_2_ breakthrough and poor sweep efficiency during CO_2_ flooding. CO_2_ Huff-n-Puff operation might overcome this issue. Hence, more studies are needed to understand this operation in detail. Based on our knowledge, there are no published studies to date that focused on modeling complex fracture networks and natural fractures explicitly and investigating their effects on the CO_2_-EOR effectiveness in multiple horizontal wells.

Numerical modeling of the well performance from tight oil reservoirs based on explicitly handling the complex fracture networks remains a challenging topic. Although significant attempts have been focused on developing unstructured grids method to model the complex fracture networks^25^,^26^, it is still limited in field-scale application due to its complexity in gridding and large computational demand, especially in the simulation of CO_2_-EOR process using compositional numerical models with multiple components. Accordingly, an efficient approach to simulate CO_2_-EOR process from tight oil reservoir with complex fracture geometry is still lacking in the petroleum industry. In this study, we introduced an embedded discrete fracture model (EDFM), which was originally proposed by Li and Lee^27^. Further extensions have been done by Moinfar *et al*.^28^ and Cavalcante Filho *et al*.^29^. Based on the EDFM, we can modify the compositional reservoir simulator in a non-intrusive manner to accurately and efficiently handle the complex fracture geometry^30^. We verified the EDFM methodology for simple hydraulic fractures against the traditional local grid refinement (LGR) approach^31^.

In this study, we first built a compositional numerical model, including four hydraulic fractures within a single stage based on the actual fluid and reservoir properties of the Bakken Formation. Four case studies with different fracture geometries from simple to complex were modelled using the EDFM. In addition, we evaluated the impacts of different fracture geometries on the well performance of CO_2_ Huff-n-Puff after three years of primary production. Subsequently, we extended the model to include two wells, each well has four fractures within single stage, to simulate CO_2_ flooding. Finally, we built a field-scale reservoir model, including two horizontal wells to study the effects of complex non-planar hydraulic fractures and natural fractures on the well performance of both CO_2_ injection scenarios under two different reservoir permeabilities of 0.01 md and 0.1 md. This work provides critical insights about the effect of fracture complexity on the well performance of CO_2_ Huff-n-Puff and CO_2_ flooding in tight oil reservoirs.

## Results

### Case Studies of CO_2_ Huff-n-Puff

We performed four case studies for CO_2_ Huff-n-Puff simulation by considering different fracture geometries within a single stage including four hydraulic fractures.

- Case 1: **Planar fractures** (Note that case 1 is considered as reference case)
- Case 2: **Diagonal fractures**
- Case 3: **Reoriented fractures**
- Case 4: **Fracture networks**

These cases consider different fracture geometries with different degrees of complexity, such as the striking angle between horizontal well and fractures, irregular fracture length of individual fracture, and the creation of fracture networks. These cases were used to quantify the effect of the increasing fracture complexity on the well performance for primary production and Huff-n-Puff scenario.

#### Case 1: Planar fractures

**Figure 1:**
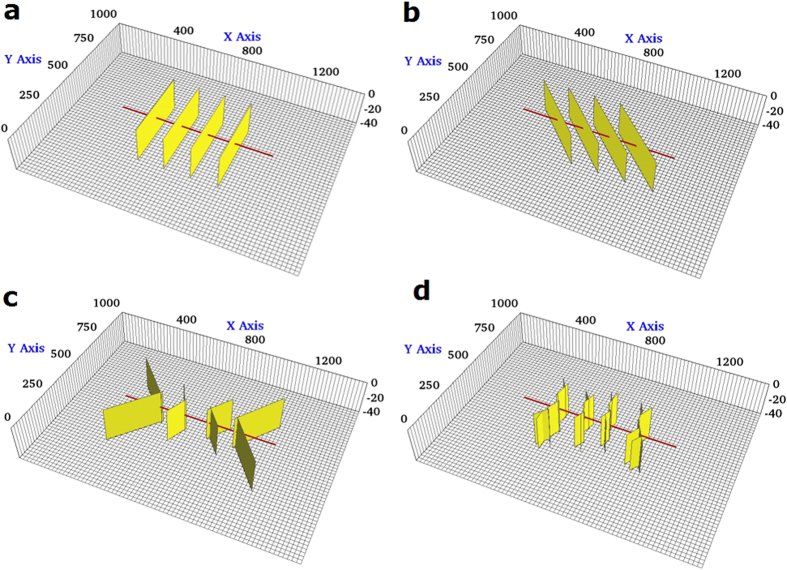
Illustration of fractures geometries evaluated for the Huff-n-Puff scenario. (**a**) Case 1: Planar fractures. (**b**) Case 2: Diagonal fractures. (**c**) Case 3: Reoriented fractures. (**d**) Case 4: Fracture networks. The yellow planes represent the fractures and the red line represents horizontal wellbore.

**Figure 2:**
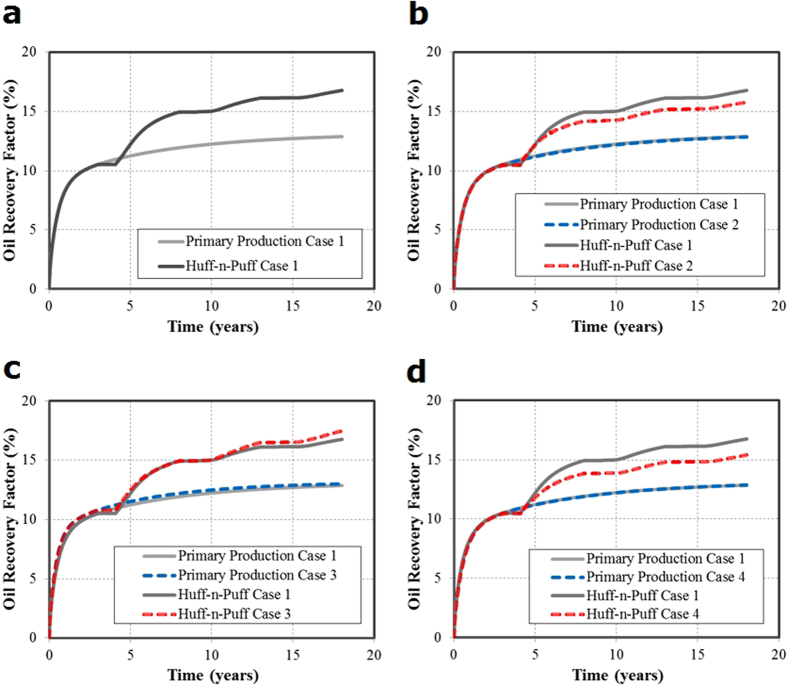
Comparison of the oil recovery factors. (**a**) Oil recovery factor for primary production and Huff-n-Puff scenario for planar fractures. (**b**) Comparison between Case 1 and Case 2. (**c**) Comparison between Case 1 and Case 3. (**d**) Comparison between Case 1 and Case 4.

This case presents four simple planar fractures orthogonal to the horizontal wellbore. All fractures have the same properties (fracture half length: 210 ft, fracture height: 40 ft and fracture conductivity: 50 md-ft). Figure 1(a) shows the projection of the aforementioned fractures. Figure 2(a) shows the comparison of oil recovery factor between the primary production and Huff-n-Puff scenario. It can be seen that the difference of the oil recovery factor at the end of simulation period is around 3.9%. As a reference case, this model was used to evaluate the impact of various cases on the well performance.

#### Case 2: Diagonal fractures

The first degree of complexity considered in this study was the orientation of the fractures with respect to the horizontal wellbore. In case 2, four fractures from an angle of 45° with the wellbore, as shown in Fig. 1(b). The other fracture properties are kept the same as case 1. Although the distance between perforation clusters is the same as case 1, the orthogonal fracture distance between two neighboring fractures was reduced to from 140 ft to 99 ft due to the change of fracture orientation. Figure 2(b) compares the difference of the oil recovery factor between cases 1 and 2. As shown, the primary oil recovery without CO_2_ injection is almost the same. However, the incremental oil recovery factor of case 2, which is around 2.9%, is less than case 1 with 3.9%. This difference is due to the changes in CO_2_ molecule distribution around the fractures. There is a lower production interference between the fractures in case 1 than in case 2, resulting in a less effective CO_2_ Huff-n-Puff for case 2. The increase in the interference between the fractures of case 2 is expected since the orthogonal distance between the fractures decreases.

#### Case 3: Reoriented fractures

Case 3 represents four non-planar fractures with outer fractures longer than inner fractures, as shown in Fig. 1(c). Each fracture is composed of two fracture segments with an orientation of 45° and 135° or 135° and 45° for the upper and lower segment, respectively. The fracture half-length for outer and inner fractures is 295 ft and 125 ft, respectively. It should be noted that the total fracture length, i.e. the summation of the fracture segments, remains the same as cases 1 and 2. Figure 2(c) compares the difference in the oil recovery factor between cases 1 and 3. As shown, there is a small difference of primary production. Nevertheless, when compared to case 1, the incremental oil recovery factor of case 3 increases from 3.9% to around 4.5%. Again, this improvement is associated with the interference between the fractures. In this case, the Huff-n-Puff is more effective than case 1 because the outer fractures have a longer length and less interference with the inner fractures.

#### Case 4: Fracture networks

The last case of fractures analyzed represents four systems of fracture networks created around each perforation cluster, as shown in Fig. 1(d). The fracture networks are composed of several segments that intersect each other. Similar to previous cases, the total fracture length of all segments is equivalent to the planar fractures of case 1. Case 4 represents a more realistic fracture geometry. Figure 2(d) compares the difference of the oil recovery factor between cases 1 and 4. In this case the incremental oil recovery is only 2.55%, which is lower than the other three cases evaluated. The Huff-n-Puff stimulated area for case 4 seems to be lower than the previous cases as a result of more serious interference between the fractures. In order to quantitatively measure the interference degree, we calculated the CO_2_-contacted area from the global CO_2_ molecule distribution maps, which is defined as the area with a CO_2_ mole fraction higher than 5%. The comparison of CO_2_-contacted area in each case is summarized in the last column of Table 1.

In addition, Table 1 lists the oil recovery factor (RF) for primary production and the incremental RF after Huff-n-Puff for each case. The results show small differences of primary production, but significant changes after Huff-n-Puff for different fracture geometries. We can notice that there is a consistent reduction in the incremental RF of CO_2_ Huff-n-Puff as the fractures become shorter and closer to each other, and the CO_2_-contacted area decreases as a result of the fracture interference. Case 4, which has the highest fracture complexity, has the lowest incremental oil recovery. This shows that characterizing the actual fracture geometry and accurately modeling the well performance from this geometry play an important role in the estimation of the additional oil recovery after the Huff-n-Puff stimulation. The use of simple planar fractures to simplify the complex fractures might overestimate the CO_2_-EOR effectiveness.

### Case Studies of CO_2_ Flooding

**Figure 3:**
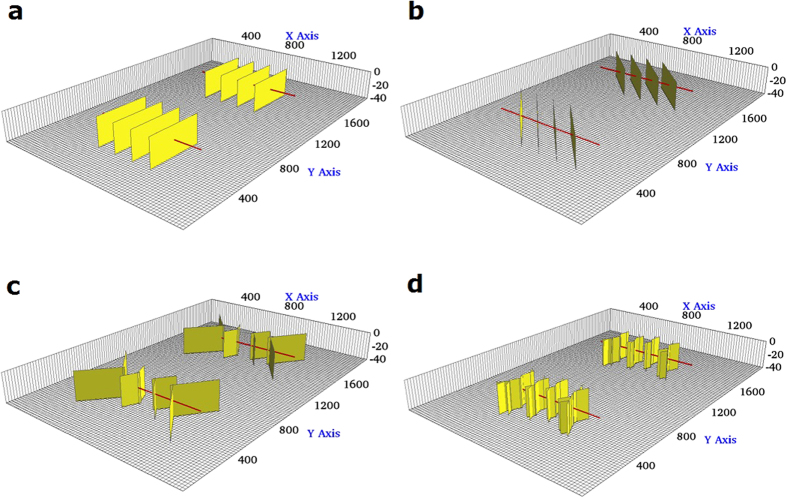
Illustration of fractures geometries evaluated for the flooding scenario. (**a**) Case 1: Planar fractures. (**b**) Case 2: Diagonal fractures. (**c**) Case 3: Reoriented fractures. (**d**) Case 4: Fracture networks. The yellow planes represent the fractures and the red line represents horizontal wellbores.

For the evaluation of CO_2_ flooding scenario, the same single-stage fracture geometries as CO_2_ Huff-n-Puff scenario were considered, as shown in Fig. 3. For these case studies, the simulation model was extended to include two horizontal wells with identical fracture geometries. The reservoir and fracture properties remain the same as those mentioned in the previous section. The distance between two wells is fixed at 1,020 ft for each case. The CO_2_ flooding scenario considers an initial period of primary production of 3 years for both wells. After that, one of the producing wells is converted to injector and used for CO_2_ injection until the end of the production time (18 years in total). The cumulative CO_2_ injection is comparable to the amount of the Huff-n-Puff scenario. The primary production simulated without CO_2_ injection is used to measure the incremental oil recovery after CO_2_ flooding.

**Figure 4:**
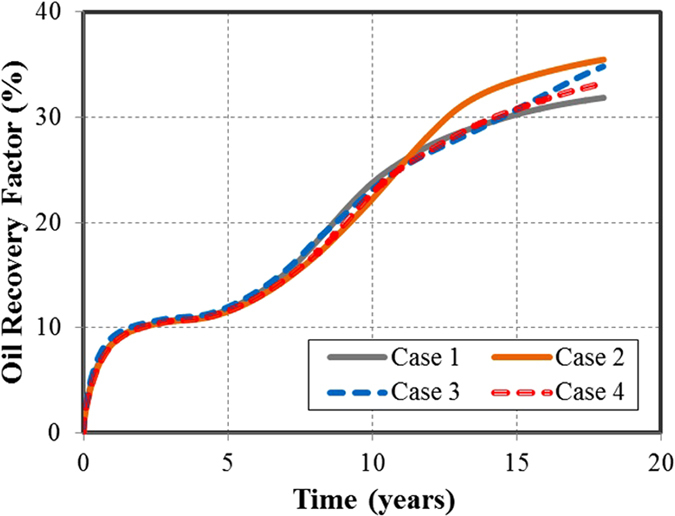
Comparison of oil recovery factor curves for four cases under CO_2_ flooding scenario.

Figure 4 compares the oil recovery factor curves for these four cases. Table 1 summarizes the oil RF of primary production and incremental RF after CO_2_ flooding and CO_2_-contacted area in each case at the end of production. As it can be seen, the incremental RF of cases 1 and 4 is lower than that of cases 2 and 3. It can be noticed from the results that there is not a direct relationship between the increase in fracture complexity and the CO_2_ flooding effectiveness. However, the incremental RFs are still in agreement with the area contacted by the CO_2_ injected, even though there are some small divergences as it can be observed in cases 2 and 3. Case 2 shows a higher recovery than case 3 but it has a slightly lower CO_2_ contacted area. Nonetheless, this can be expected since the average CO_2_ concentration in the contacted area in case 3 is higher than case 2 (0.60 and 0.55, respectively). For the flooding scenario a higher contacted area is due to the location of the fractures. More particularly, it is because of the dimensions of the cross sectional area covered by the fractures between the injector and producer. It can be clearly observed in the Fig. 3 that cases 2 and 3 have a larger extension in the x-axis direction (717 ft and 572 ft respectively), whereas cases 1 and 4 have a smaller extension (420 ft and 529 ft respectively). A longer extension of the fractures in the x-axis direction allows a higher CO_2_-contacted area and therefore a higher incremental oil recovery. For the CO_2_ flooding, the fracture geometry is also a key factor affecting the estimation of the additional oil recovery. However, the inclusion of the complex fractures for the flooding scenario, unlike the Huff-n-Puff, does not necessarily implies a negative effect in the incremental oil recovery.

### Field-Scale Case Study

This section is devoted to analyze the influences of complex fracture geometries on the well performance of CO_2_ Huff-n-Puff and CO_2_ flooding by building a realistic field-scale reservoir model. Two permeabilities such as 0.01 md and 0.1 md are considered to represent low and high permeability, which are within the range of permeability in the Bakken Formation. In addition, we also examine the effect of natural fractures on the production performance. For these purposes, the original model was extended to 5,240 ft × 2,680 ft × 40 ft (262 × 134 × 1 cells), which is able to model two horizontal wells with lateral length of 4,640 ft for each one and well spacing of 1,340 ft. Each well has 15 stages and each stage is assumed to have a single effective hydraulic fracture. The fracture spacing is 290 ft. We performed both CO_2_ Huff-n-Puff and CO_2_ Flooding simulations by considering the following cases:

- Non-planar fractures
- Non-planar fractures with one set of natural fractures

**Figure 5:**
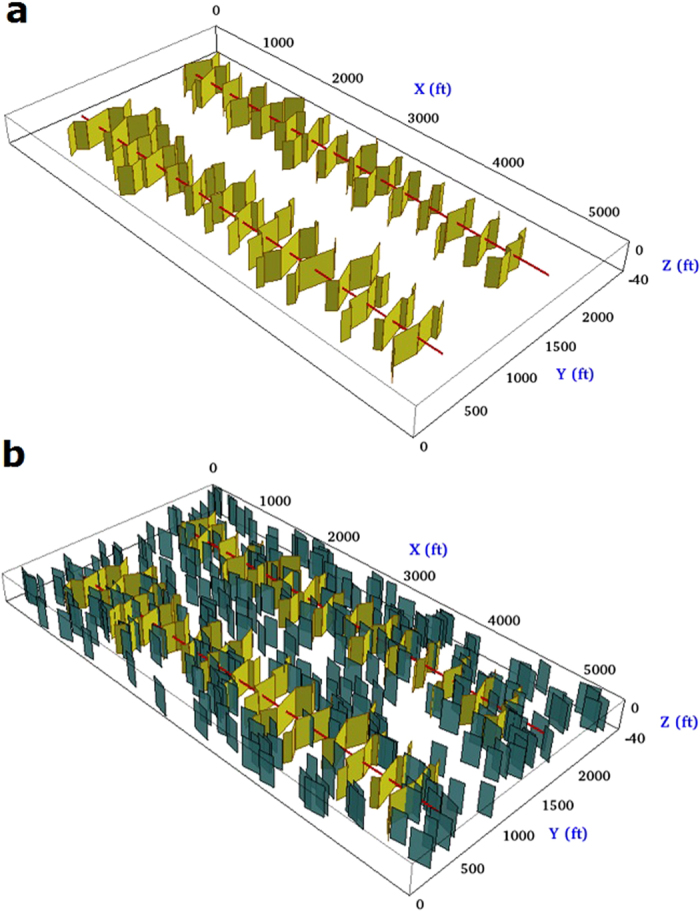
Illustration of non-planar fracture geometry used for the field-scale simulation, taking into account the presence of natural fractures. (**a**) Non-planar fractures with irregular dimensions (**b**) Non-planar fractures with a set of natural fractures parallel to the horizontal wellbore.

In the first case, shown in Fig. 5(a), all the fractures are non-planar and have different dimensions. The fracture half-length varies from 194 to 445 ft. The fracture height and conductivity used are 40 ft and 50 md-ft, respectively. Both fracture geometries have the same fracture spacing of 290 ft.

One additional cases were set in order to evaluate the impact of the natural fractures on the performance of the CO_2_ Huff-n-Puff and CO_2_ flooding, as presented in Fig. 5(b). This case includes a set of 300 natural fractures, which are randomly distributed with the assumption that their orientations are parallel to the horizontal wellbore. These natural fractures have a length ranging from 100 to 200 ft, a height of 40 ft, and a fracture conductivity of 5 md-ft. The combination of modelling complex non-planar hydraulic fractures and natural fractures permits to model more realistic fracture networks.

#### CO_2_ Huff-n-Puff field-scale study

**Figure 6:**
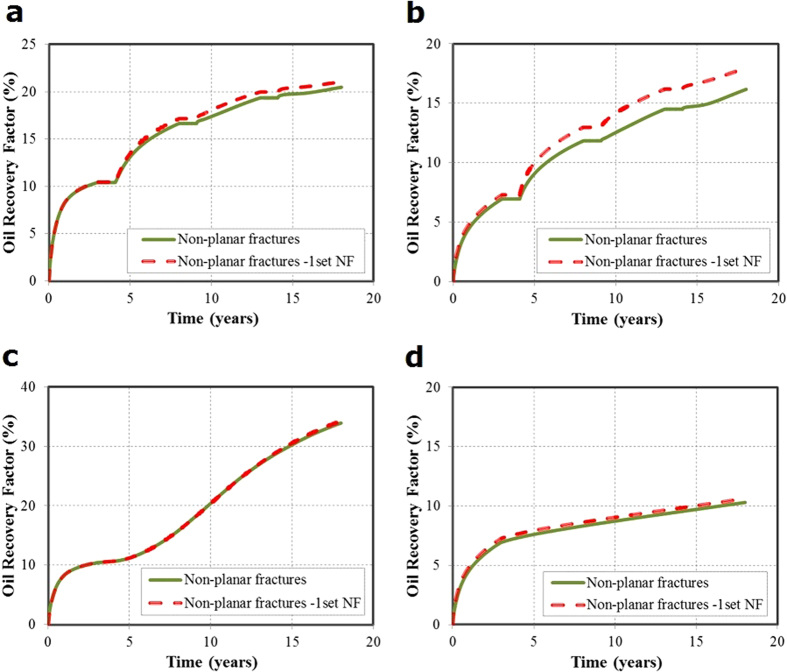
Oil recovery factors for CO_2_ Huff-n-Puff and CO_2_ flooding under matrix permeability values of 0.1 md and 0.01 md. (**a**) Oil recovery factors of CO_2_ Huff-n-Puff for permeability of 0.1 md. (**b**) Oil recovery factors of CO_2_ Huff-n-Puff for permeability of 0.01 md. (**c**) Oil recovery factors of CO_2_ flooding for permeability of 0.1 md. (**d**) Oil recovery factors of CO_2_ flooding for permeability of 0.01 md.

We performed the simulations for CO_2_ Huff-n-Puff scenario and the results for both high and low permeabilities are shown in Fig. 6. For the cases evaluated there was no significant differences in the oil recovery factor during primary production regardless of high or low permeability. For the high permeability of 0.1 md, the maximum difference between the cases is less than 0.05%, and for the low permeability of 0.01 md the difference is about 0.4%. Similarly, the enhanced oil recovery at the high permeability, shown in Fig. 6(a), reflects a small impact. The incremental oil recovery factor is about 7.7% for the non-planar fractures when compared to the primary production. The previous results observed in the single stage study showed a bigger difference because the fracture spacing used was 140 ft, which is lower than the fracture spacing of 290 ft used for the field case. Then, the fracture interference decreases with increasing fracture spacing and there is only a small difference of 0.6% in the incremental oil recovery factor between the planar and non-planar fracture geometries. In addition, the effect of natural fractures is also small. The incremental oil recovery factor is 8.2% for the additional set of natural fractures.

On the other hand, the results at low permeability of 0.01 md, presented in Fig. 6(b), show a significant difference in the incremental oil recovery factor when the natural fractures are included. The incremental recovery factor in the case with one set of natural fracture increases from 5.2% to 6.8% when compared to the non-planar fractures without natural fractures. Hence, the presence of natural fractures significantly impacts the well performance of CO_2_ Huff-n-Puff under the low permeability of 0.01 md. It can be implied that the natural fractures should be correctly included in the numerical model for simulating CO_2_ Huff-n-Puff in some tight formations with low permeability and high density of natural fractures.

**Figure 7:**
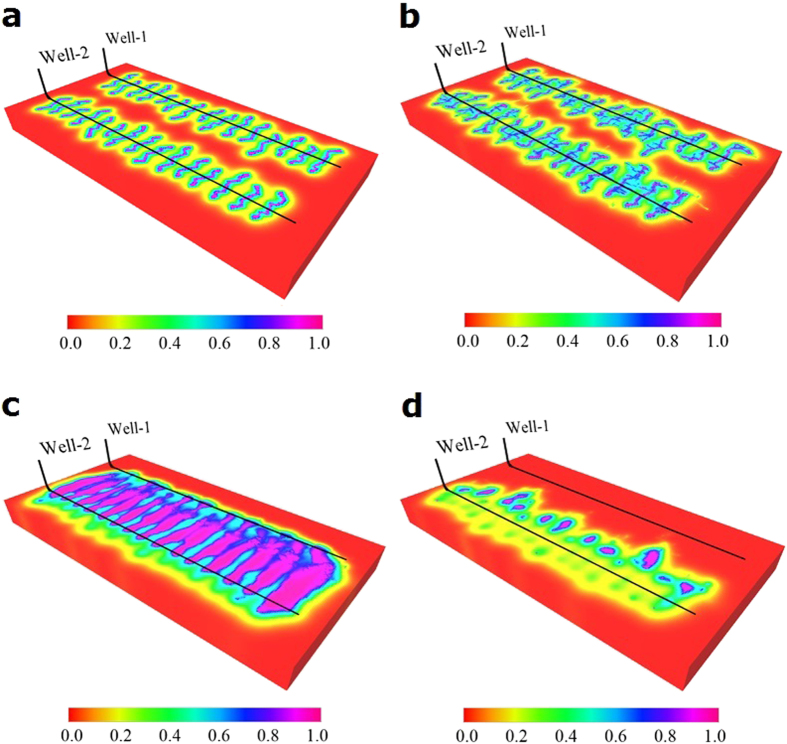
Global CO_2_ molecule distribution for Huff-n-Puff and flooding scenarios with and without natural fractures under matrix permeability values of 0.1 md and 0.01 md. (**a**) Global CO_2_ molecule distribution for Huff-n-Puff after one year of CO_2_ injection under permeability of 0.01 md without natural fractures (**b**) Global CO_2_ molecule distribution for Huff-n-Puff after one year of CO_2_ injection under permeability of 0.01 md with natural fractures (**c**) Global CO_2_ molecule distribution for flooding after 15 years of CO_2_ injection under permeability of 0.1 md. (**d**) Global CO_2_ molecule distribution for flooding after 15 years of CO_2_ injection under permeability of 0.01 md.

The previous observations can be verified by comparing CO_2_ molecule distribution maps, as shown in Fig. 7. The molecular fraction distribution maps of CO_2_ presented in Fig. 7(a,b) show the concentrations of CO_2_ for the non-planar fractures with and without the set of natural fractures under low permeability of 0.01 md. It can be clearly noticed that under low permeability, the non-planar fracture geometry with one set of natural fractures has a higher concentration of CO_2_ compared to without natural fractures especially for the values of around 0.6 displayed in cyan. Hence, the CO_2_ distribution maps show that the performance of CO_2_ Huff-n-Puff under the low permeability is more sensitive to the presence of natural fractures.

#### CO_2_ flooding for field-scale study

Similarly, we performed the simulations for CO_2_ flooding scenario and the results for both high and low permeabilities are shown in Fig. 6. The effect of the natural fractures, which are parallel to the horizontal wellbore, is not significant and the incremental oil recovery factor is similar to that obtained from the non-planar fractures without natural fractures (21.5% and 21.2%, respectively). Regarding the comparison for the low permeability of 0.01 md and high permeability of 0.01 md, Fig. 7(d) show that the flooding scenario for low permeability is not favorable because the injected CO_2_ cannot reach the production well (Well 1) due to the poor connectivity between two wells, unlike the case of high permeability shown in Fig. 7(c). The final recovery factors are lower than the primary production, which includes the production of both wells. Hence, we can notice that the presence of natural fractures has a higher importance in the production response of the CO_2_-EOR in the cases with low permeability, especially for the Huff-n-Puff scenario.

## Discussion

The reservoir model built allowed to evaluate the effects of the complex fracture geometries on the well performance of CO_2_-EOR using the EDFM approach. From the presented results we observed the fracture geometry has an important effect on the Huff-n-Puff treatment. The CO_2_ Huff-n-Puff is quite sensitive to the fracture geometry because its effectiveness is mainly determined by the fracture interference. As we have mentioned previously, the interference between fractures increases as the fracture segments become closer and shorter, which are the cases of complex fractures and fracture networks. Therefore, the expected incremental recovery decreases. Most of the models reported in the scientific literature use planar fractures, but if this model is used for the evaluation of CO_2_ Huff-n-Puff, the incremental recovery will be certainly overestimated.

For the CO_2_ flooding scenario the non-planar fractures do not show a negative effect on the EOR performance. It can improve the effectiveness of the flooding if the location of the fractures turns into a higher contacted area, which depends on the configuration of the fractures. The CO_2_ flooding is less sensitive to the fracture geometry itself, but it is affected by the extension of the injection front developed by such fracture geometry.

Although there has been an improvement in the techniques of fracture diagnostics, the actual fracture geometry is still not well understood. The fracture geometry obtained from such techniques is non-unique and the uncertainty in the fracture geometry should be taken into account for the evaluation of EOR treatments. For an adequate estimation, it would be useful to make a sensitivity analysis of all the possible fracture geometries to obtain a range of values for the incremental recovery, especially for the CO_2_ Huff-n-Puff. For the CO_2_ flooding the focus should be on the extension of the cross-sectional area of the gas front.

The case studies performed with natural fractures present in the reservoir show that the improvement of the CO_2_ Huff-n-Puff is only significant for the low permeability of 0.01 md. This increment in the oil recovery responds to the increase in the CO_2_ contacted area. In the cases of high permeability the contacted area was already high and the natural fractures did not affect it significantly. However, The CO_2_ flooding is not favorable for low permeability of 0.01 md due to poor connectivity between two wells, while it is suitable for high permeability of 0.1 md. The CO_2_ Huff-n-Puff performs better than the CO_2_ flooding for low permeability of 0.01 md.

## Methods

### Embedded Discrete Fracture Model

EDFM is an efficient model to handle complex fracture geometry in reservoir simulators. Using this method, fractures can be discretized into small fracture segments with matrix cell boundaries. Virtual cells are created to represent these fracture segments. This method can be applied in traditional reservoir simulators in a non-intrusive manner by appending cells in the grid domain and adding non-neighboring connections (NNCs) for these cells to account for the mass transport associated with fractures, including the flow between matrix and fractures, flow inside an individual fracture, and flow between intersecting fractures. Through transmissibility factors between corresponding cells, the volume flow rate of phase *l* between cells in a NNC pair is:


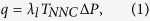


where *λ*_*l*_ is the relative mobility of phase *l*, *T*_*NNC*_ is the NNC transmissibility factor, and Δ*P* is the potential difference between the cells.

For the calculation of transmissibility factors, generally, *T*_*NNC*_ can be expressed as


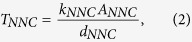


where *k*_*NNC*_, *A*_*NNC*_, and *d*_*NNC*_ are the permeability, contact area, and distance associated with this connection, respectively.

For matrix-fracture connection, in Eq. 2, *k*_*NNC*_ is the matrix permeability in the direction perpendicular to the fracture plane, *A*_*NNC*_ is the area of the fracture plane inside the matrix block, *d*_*NNC*_ is the average normal distance from matrix block to fracture plane.

For connections between fracture segments, *k*_*NNC*_ is calculated as an average of fracture permeability, *A*_*NNC*_ is the common area between fracture segments, and *d*_*NNC*_ is the distance between centroids of the fracture segments. More details of the calculation can be found in Xu^30^.

In addition, the fracture-wellbore intersections are modeled as effective well indices in the EDFM. The effective well index in the EDFM can be calculated as^30^


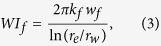



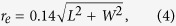


where *k*_*f*_ is the fracture permeability, *w*_*f*_ is the fracture aperture, *L* and *W* are the length and height of the fracture segment, respectively. Eqs 3 and 4 can be derived by replacing the dimensions and permeability of the gridblock in Peaceman’s model with those of the fracture segments.

By discretizing large fractures into interconnected small fractures, the EDFM has been proven to be effective in modeling complex hydraulic fracture geometries such as non-planar fractures and fractures with variable width^30^. Furthermore, the computational performance of the EDFM in traditional simulators was also verified through detailed comparison with LGR model. In this study, we first present a case study to verify the EDFM with LGR model, then for other cases, we apply the EDFM method in the simulator to simulate complex fracture geometries.

### Reservoir Simulation Model

A compositional numerical reservoir model with single fracture stage was built based on the typical reservoir and fracture properties of the Middle Bakken Formation using a reservoir simulator^32^. A Cartesian grid system was used, which consists of 70 grids in *x* direction, 51 grids in *y* direction, and 1 grid in *z* direction. The dimension of the reservoir model is 1,400 ft × 1,020 ft × 40 ft, which corresponds to length, width, and thickness, respectively. A porosity of 7% and a permeability of 0.1 md were considered which are the typical reservoir and fracture properties from the Middle Bakken Formation^33^,^34^. The study considers homogeneous and isotropic reservoir. The relative permeability curves including the water-oil relative permeability and liquid-gas relative permeability are from our previous study^12^, which were generated based on history matching with a field production well from the Middle Bakken Formation. More detailed information can be found in the Supplementary Information document. The molecular diffusion is one the main mechanisms of EOR in tight reservoirs, since the gravity and viscous forces are not dominant because of the low permeability. The Sigmund method was used to model the physical mechanism of CO_2_ molecular diffusion^35^,^36^. The CO_2_ molecular diffusion coefficient in the oil phase was set at 0.001 cm^2^/s in this study. More details about the CO_2_ molecular diffusion effect can be found in the work by Yu *et al*.^13^.

The oil composition used in the model and the main oil properties were taken from the study of crude oil composition for the Middle Bakken Formation by Yu *et al*.^13^, which were determined based on matching the key fluid properties reported for the Bakken formation in the scientific literature^4^,^33^. The Peng-Robinson equation of state^37^ was used and the model considers seven pseudo-components, i.e., CO_2_, N_2_, CH_4_, C_2_-C_4_, C_5_-C_7_, C_8_-C_9_, C_10+_, with the corresponding molar fractions of 0.02%, 0.04%, 25%, 22%, 20%, 13%, and 19.94%, respectively. The phase behavior of the crude sample was modelled using CMG-WinProp^38^. The final determined oil properties are: oil gravity is 42° API, GOR is 1,000 SCF/bbl, bubble point is 2,000 psi, oil formation factor is 1.6 STB/bbl. It is important to point out that these oil properties are within the reasonable range of typical oil properties of the Middle Bakken Formation. In addition, the MMP was calculated as 3,334 psi, which was also in the range reported by other experiments^6^.

The initial conditions considered for the simulation were an initial pressure of 8,000 psi for a reservoir depth of 11,000 ft, an initial oil saturation of 80%, an initial water saturation of 20%, and a reservoir temperature of 240 °F. The initial pressure does reflect an overpressured reservoir, as it is usually observed in tight and shale oil fields^39^. The overpressure is related to the cracking of the organic material and the increase in fluid volume. Also, it is an indicator of the maturity of the shale formation and correlated to a higher oil production. The model uses a no-flow boundary condition. The calculated original oil in place was 3.746 MMSTB. The bottomhole pressure (BHP) was used as the primary constraint for the reservoir simulation and a value of 1,800 psi was set for the production wells. For the injection wells, two constraints were used. The first one is a constant injection rate. 1,000 MSCF/D and 2,500 MSCF/D were used for the flooding case and Huff-n-Puff case, respectively. Both cases have the same cumulative injection volume. The second one is a maximum BHP of 8,000 psi. The BHP constraint and injection rates were set in such a way that the MMP could be reached after a short time of injection and the pressure is maintained then above the MMP to achieve the miscible condition during the whole simulation period. If the miscible condition is not achieved, the CO_2_ Huff-n-Puff scenario will show a poor performance because the presence of multiphase flow results in a decrease in relative permeability of oil. The optimized production schemes reported in the literature performed also the CO_2_ injection under miscible conditions^40^,^41^.

## Additional Information

**How to cite this article**: Zuloaga-Molero, P. *et al*. Simulation Study of CO_2_-EOR in Tight Oil Reservoirs with Complex Fracture Geometries. *Sci. Rep*. **6**, 33445; doi: 10.1038/srep33445 (2016).

## Supplementary Material

Supplementary Information

## Figures and Tables

**Table 1:** Summary of oil recovery factor (RF) of primary production and incremental RF after CO_2_ Huff-n-Puff and flooding for the different cases and CO_2_-contacted area for each case.

CO_2_ Huff-n-Puff				
Case Number	RF Primary Production (%)	RF Huff-n-Puff (%)	Incremental RF (%)	CO_2_-Contacted Area (×1000 ft^2^)
Case 1	12.9	16.8	3.9	170.9
Case 2	12.8	15.7	2.9	160.9
Case 3	12.9	17.4	4.5	180.2
Case 4	12.8	15.4	2.6	152.6
**CO_2_ Flooding**				
**Case Number**	**RF Primary Production (%)**	**RF Flooding (%)**	**Incremental RF (%)**	**CO_2_-Contacted Area (×1000 ft^2^)**
Case 1	12.9	31.8	18.9	1054.9
Case 2	12.9	35.4	22.5	1151.4
Case 3	13.0	34.8	21.8	1187.2
Case 4	12.9	32.9	20.0	1095.5
