# Supplementary material for: Simulation Study of CO2-EOR in Tight Oil Reservoirs with Complex Fracture Geometries
Source: Sci Rep. 2016 Sep 15;6:33445. doi: 10.1038/srep33445 (PMC5024126; doi:10.1038/srep33445)
Supplement: Supplementary Information [file srep33445-s1.pdf]

Supplementary information to:

Simulation Study of CO_2-EOR in Tight Oil Reservoirs with Complex Fracture Geometries

Authors:

Pavel Zuloaga Molero[1], Wei Yu[2,*], Yifei Xu[1], Kamy Sepehrnoori[1], Baozhen Li[3]

Affiliations:

[1]Department of Petroleum and Geosystems Engineering, University of Texas at Austin, Austin, TX, 78712, USA.

[2]Department of Petroleum Engineering, Texas A&M University, College Station, TX, 77843, USA.

[3]CNOOC Research Institute, Beijing, 100027, China.

*Corresponding author

Contact Information of Corresponding Authors:

Correspondence and requests for materials should be addressed to W. Yu (yuwei127@tamu.edu, +1 512 574 0080)

Supplementary Material and Methods

Relative Permeability Curves. The relative permeability curves used in the reservoir model were obtained from the history matching of a well production from the Bakken Field. The endpoints and exponents from Corey correlation were used as tuning parameters. The final relative permeability curves are shown in the **Supplementary Figure 1**.

Verification of EDFM. For the verification purpose, we only model four simple planar hydraulic fractures with fracture conductivity of 50 md-ft, fracture length of 210 ft, and fracture height of 40 ft. The comparison of grid blocks used to model hydraulic fractures between EDFM and LGR is displayed in the **Supplementary Figure 2**. As shown, the LGR approach has more grid blocks to handle fractures than the EDFM approach. The **Supplementary Figure 3** presents the comparison of oil recovery factor using LGR and EDFM to model four hydraulic fractures in a single stage for a period of primary production (4 years) and three consecutive cycles of CO_2 Huff-n-Puff. Each cycle contains 1 year of injection, 1 month of soaking period, and 4 years of production. As it is observed, a good agreement was obtained and therefore the EDFM methodology can be applied to properly model the CO_2 injection in tight oil reservoirs with hydraulic fractures.

***Sensitivity analysis of vertical layering.*.** To evaluate the impact of segregation, we performed two case studies based on the CO_2 flooding with different vertical layering: one with single layer and another one with five layers. **Supplementary Figure 4** presents the comparison results of well performance. It can be seen there is

not noticeable differences between the single layer and multiple layers. However, it is important to notice that this is only true for small thickness values. As the thickness increases the model becomes more sensitive to the number of layers.

Supplementary Figures

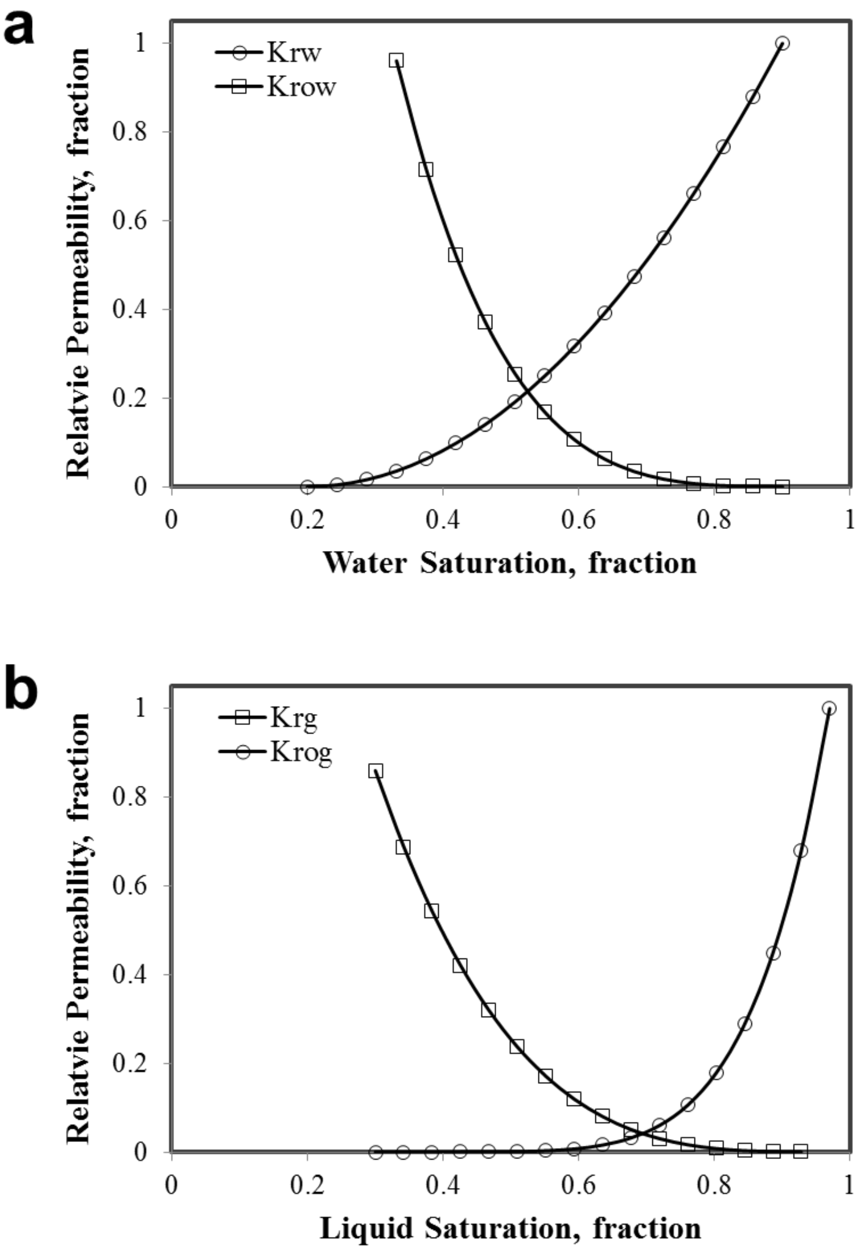

Supplementary Figure 1. Relative permeability curves for a good history match. (a) Water-oil relative permeability curve . **(b)** Liquid-gas relative permeability curve.

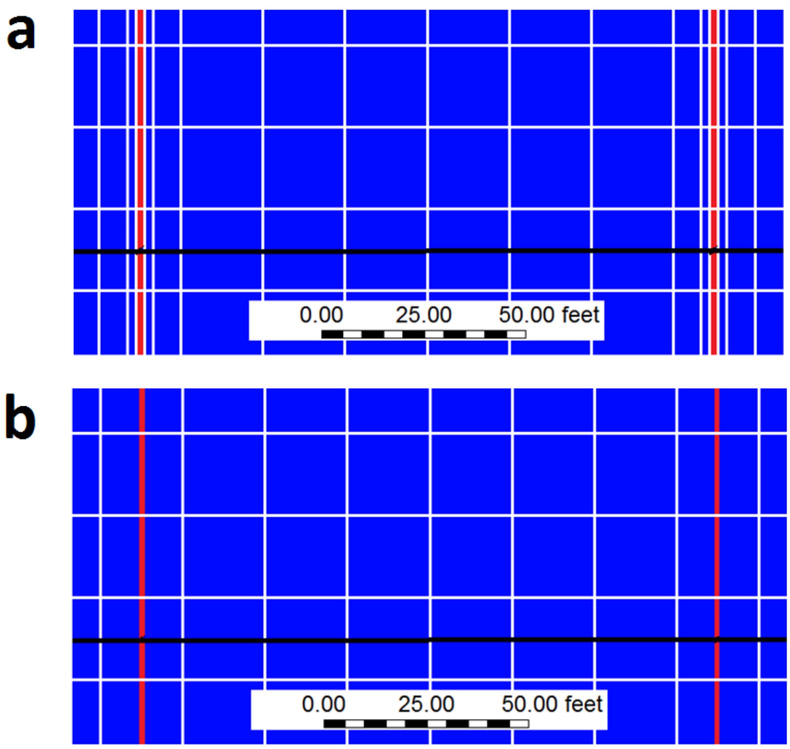

Supplementary Figure 2. Comparison of grid blocks to model hydraulic fractures using the methodologies of LGR and EDFM (The red lines represent fractures). **(a)** Grid blocks to model fracture using LGR. **(b)** Grid blocks to model fracture using EDFM

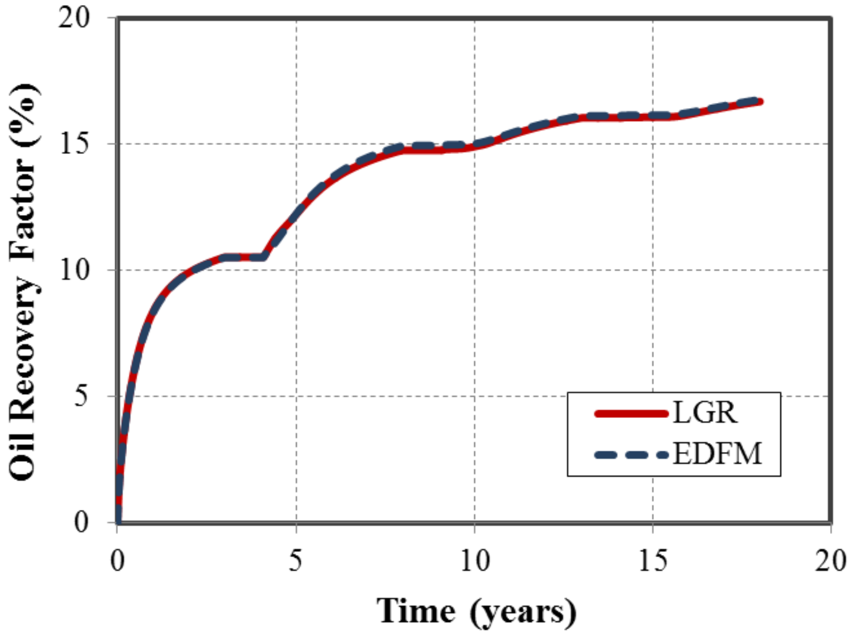

Supplementary Figure 3. Comparison of oil recovery factor for four years of primary production and three cycles of Huff-n-Puff using LGR and EDFM methodologies.

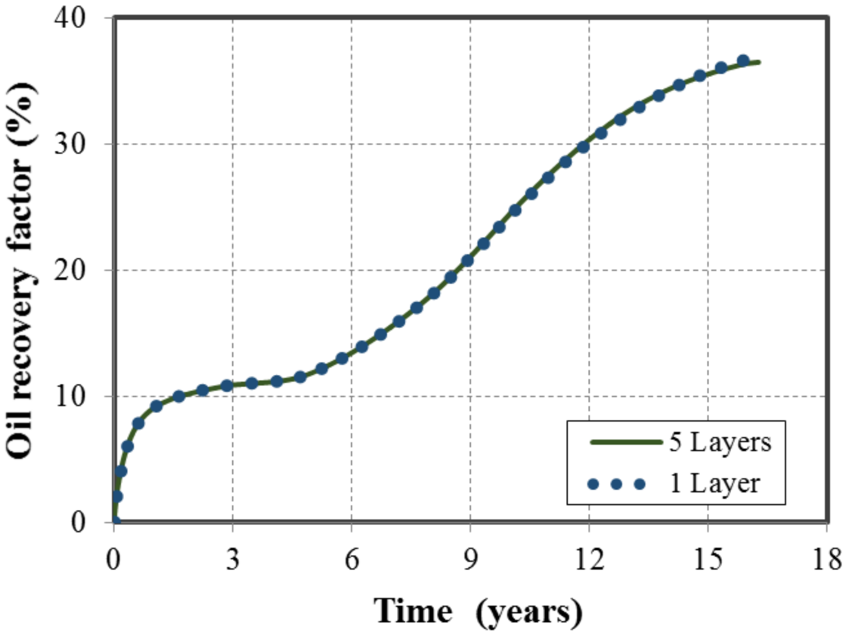

Supplementary Figure 4. Sensitivity analysis of the number of vertical layers on well performance for the entire production lifetime.
